# Optimizing the implementation of case-area targeted interventions during cholera outbreaks with context-specific delivery mechanisms

**DOI:** 10.1371/journal.pntd.0013534

**Published:** 2025-09-23

**Authors:** Jessica Dunoyer, Ruwan Ratnayake, Sandy Moore, Gregory Bulit, Samuel Beaulieu, Christophe Valingot, Pierre-Yves Oger, Bertrand Sudre, Daniele Lantagne, Nicola Desmond

**Affiliations:** 1 London School of Hygiene and Tropical Medicine, London, United Kingdom; 2 Prospective and Cooperation, Marseille, France; 3 United Nation Children’s Fund, New York, United States of America; 4 Tufts University, Friedman School of Nutrition, Boston, Massachusetts, United States of America; Yale University School of Medicine, UNITED STATES OF AMERICA

## Abstract

Cholera, a severe fecal-oral disease, disproportionately affects the poorest communities who lack access to safe water and sanitation. Individuals living in the same household, or within a few hundred meters, of a patient are at increased risk of infection. Thus, during cholera outbreaks, targeted response strategies, such as case-area targeted interventions (CATIs), provide health (e.g., vaccination and antibiotic prophylaxis) and water, sanitation, and hygiene services for affected households and at-risk neighbors living in a defined ring. Previous research on CATIs has focused on impact and effectiveness, and less on implementation processes. As cholera outbreaks occur in diverse settings with differentiated challenges, we investigated how CATI and CATI-like mechanisms can be best used and adapted. Drawing on 43 peer-reviewed articles and gray literature sources retrieved through a narrative review, and 15 key informant interviews conducted using a snowball sampling approach, we identified 27 CATI or CATI-like experiences across 15 countries in Africa, Asia, the Caribbean, and Middle East between 2004 and 2024. Four delivery mechanisms were identified: CATI, pre-CATI, case-cluster, and health-facility-based interventions (HBI). Challenges to implementation included: delays in response; difficulty accessing populations; resource shortages to initiate, maintain, or scale up response; overwhelmed response capacity; limited skills and knowledge; low uptake and acceptance; weak coordination; poor reporting and monitoring; and sustainability concerns. Implementers adapted delivery to overcome challenges, particularly in outbreaks with high case-loads and in insecure and hard-to-reach contexts by ensuring readiness and early activation, strengthening local actors’ capacity, optimizing resources, adjusting ring sizes, and prioritizing cases. Based on these results, we developed a practitioner-centered framework to optimize programmatic implementation through context-specific delivery mechanism and ultimately decrease cholera incidence.

## Author summary

Cholera, a life-threatening disease transmitted through contaminated food and water, disproportionately impacts poor communities with limited access to safe water and sanitation. People living near cholera patients face a heightened risk of infection, prompting the development of targeted responses to interrupt community transmission. These strategies aim to provide health, and water, sanitation, and hygiene services to affected households and neighbors. Our study identified different approaches to deliver health and WASH services, along with associated challenges and adaptations. We developed a practical framework to help implementers tailor targeted interventions to specific contexts to enable programmatic implementation success.

## Introduction

Cholera, a diarrheal disease caused by the ingestion of the bacterium *Vibrio cholerae* O1, poses a significant public health concern in low- and middle-income countries [1–3]. An estimated 1.3 to 4.0 million cholera cases, and 21,000–143,000 cholera deaths, occur each year [1]. This health threat, sometimes referred to as the “disease of poverty”, disproportionately affects the most vulnerable individuals who lack access to basic water and sanitation services [3,4]. In 2024, a cumulative total of 804,721 cholera cases and 5,805 deaths were reported across 33 countries [5]. This represents a marked increase compared to 2023: a 50% rise in cases and a 45% increase in deaths.

Cholera outbreaks tend to cluster geographically and temporally [6–9]. Household members of cholera patients face a relatively higher risk of infection [8,10]. Individuals living in close proximity to cholera cases (within a few hundred meters) are also at higher risk, especially during the first week after the cholera patient seeks treatment [6,8,11,12,13].

To control cholera, the case-area targeted intervention (CATI) approach has been developed [7]. CATIs specifically target the homes of cholera patients and their neighbors in a defined radius with water, sanitation, and hygiene (WASH) and health interventions, including administration of oral cholera vaccine (OCV) and selective antibiotic prophylaxis upon admission to cholera treatment facilities [8,14]. Although the concept of CATIs traces back to Cameroon in 2004 [8,14,15], its prominence grew after the 2010 cholera epidemic in Haiti and subsequent nationwide expansion led by UNICEF of an alert-response system in 2013 [16]. CATIs have been effective in reducing cholera incidence by significantly reducing cases (up to 76%) and shortening outbreak duration (up to 73%), with greater impact for prompt and intense response in Haiti [17]. In the Noth East Zone in Nigeria, clusters of cholera cases were fewer and smaller, occurred for shorter duration, and were less likely to reoccur in 2021 in the presence of CATIs implemented by *Solidarités International* (SI) and *Action Contre le Faim* (ACF) [18].

Similar delivery mechanisms that target areas surrounding case-households have also been developed, which are described herein as ‘CATI-like’ mechanisms. In this context, a delivery mechanism refers to the organized way in which interventions such as WASH supplies, health education, and vaccines are brought to affected populations. The case-cluster approach, which entails the delivery of targeted WASH and health interventions to households located within a cluster of cholera cases, was used in Conakry by *ACF* in 2012 [19]. In 2017, the health authorities in Kinshasa adopted a grid strategy, assigning teams to households within each grid for 14-day WASH and health responses in highly affected neighborhoods [20]. Health facility-based outreach activities were developed by *Médecins Sans Frontières* (MSF) in Haiti in 2013 [21] and in the Democratic Republic of Congo (DRC) in 2017 [22], and by *John Hopkins University* (JHU) in Bangladesh in 2013 [23], and in DRC in 2020 [24]. In the MSF-led intervention in DRC, evidence showed a dose-response relationship, with higher use of hygiene kit (soap, point-of-use water treatment, a handwashing device, and a 20-liter jerrycan) linked to a 66% lower incidence of suspected cholera and a significant reduction in water contamination across all recipients. In the JHU-led project in Bangladesh, the intervention promoted handwashing with soap and point-of-use water treatment among cholera patients and their household members. Intervention contacts had 47% fewer *Vibrio cholerae* infections than controls, were more likely to wash hands during key times (18% vs. 50%), and had a 41% reduction in very high-risk households with stored drinking water after 6–12 months [23,25]. Overall, in multiple evaluations, CATI and CATI-like mechanisms have been found effective, and are now widely promoted in cholera response [8].

In these previous studies, barriers to CATI delivery have been identified, including: 1) difficulties in accessing patient homes (e.g., due to lack of address, flooding, insecurity, or stigmatization) [8,14], 2) delayed or partial access to the case home [8,14,26], 3) difficulties in achieving a sufficient case or ring coverage (e.g., during high case load, in densely populated areas, due to resource constraints) [8,14,26], and 4) limited funding and supplies [8,14,26,27].

However, despite identifying some challenges, previous research was not comprehensive and had less focus on developing recommendations for implementation. To date, recommendations on how to implement CATI and CATI-like mechanisms in different contexts have not been summarized or published, limiting cross-learning opportunities. We believe that optimizing CATI implementation across contexts is essential to enhance response timeliness, expand coverage, increase acceptance and uptake, improve cost-efficiency, and ultimately reduce the cholera disease burden.

We aimed to synthesize available evidence on adapting CATI and CATI-like mechanisms to inform global coordination platforms developing technical guidance for targeted cholera outbreak interventions. The study addressed four key objectives: 1) describe and categorize CATIs and CATI-like mechanisms used during cholera outbreaks, 2) identify and summarize recurrent and context-specific implementation challenges, 3) review and summarize implementation adaptations to optimize delivery in different contexts, and 4) develop an implementation framework for each delivery mechanism to improve the effectiveness of the response.

## Methods

### Ethics statement

All interviewees were fully informed about the purpose of the research, their rights as participants, and measures in place to ensure confidentiality. Written informed consent was obtained from each participant before the interview. Additionally, a brief verbal reaffirmation of consent was conducted immediately before the interview, allowing participants an opportunity to ask final questions or seek clarification. To protect participant anonymity, transcripts and publications do not contain direct links to specific quotes or identifiable statements. Ethical approval for this study was granted by the LSHTM Ethics Committee (LSHTM #29997).

This research was mixed-method, including the following: 1) narrative review of peer-reviewed journal publications and gray literature published between January 1, 2000 and June 1, 2024, and 2) key informant interviews (KIIs) of researchers, cholera technical advisors, and/or implementers in five countries recently affected by cholera (DRC, Mozambique, Nigeria, Ethiopia, and Somalia). Objectives 1, 2, and 3 were investigated primarily using the narrative review, complemented by the KIIs. The narrative review and KIIs both informed development of the implementation framework for CATI and CATI-like mechanisms, tailored to different contexts (Objective 4).

### Narrative review

The objective of this narrative review was to identify and characterize the delivery mechanisms, challenges, and adaptations made in implementing CATIs during cholera outbreaks, as reported in peer-reviewed and gray literature.

We conducted a literature search in the Medline and Embase databases for articles published between January 1, 2020, and June 7, 2024, using keywords related to cholera and CATIs. The below search terms were used:

- Keyword 1: cholera; acute watery diarrhea; vibrio cholerae
- Keyword 2: case-area targeted intervention; case-area targeted response; targeted intervention; targeted response; ring intervention; rapid response; mobile response; household intervention; household response; household disinfection; home disinfection; household spraying; hospital-based intervention; health facility-based intervention; alert and response

The full search strategy is provided in S1 File. In addition, we retrieved gray literature published during the same period from organizational websites and coordination platforms (S1 Table). We also asked participants in KIIs to share relevant documentation.

To ensure completeness, we additionally included peer-reviewed articles and gray literature previously identified in two earlier systematic reviews on CATI effectiveness, which covered the periods January 1, 2009 – November 30, 2019 [14], and January 1, 2000 – April 24, 2020 [8].

### Inclusion criteria

We included records that:

- Were published in English (for peer-reviewed articles) or in French or English (for gray literature),
- Were published between January 1, 2000, and June 7, 2024,
- Described targeted interventions implemented at the case-household level during cholera outbreaks.

### Exclusion criteria

We excluded records that:

- Focused on other aspects of cholera control (e.g., general epidemiology, surveillance, vaccine development, or case management),
- Did not include implementation description or evaluation (e.g., modeling studies, policy comparisons, or guideline reviews),
- Addressed diseases other than cholera.

**Fig 1:**
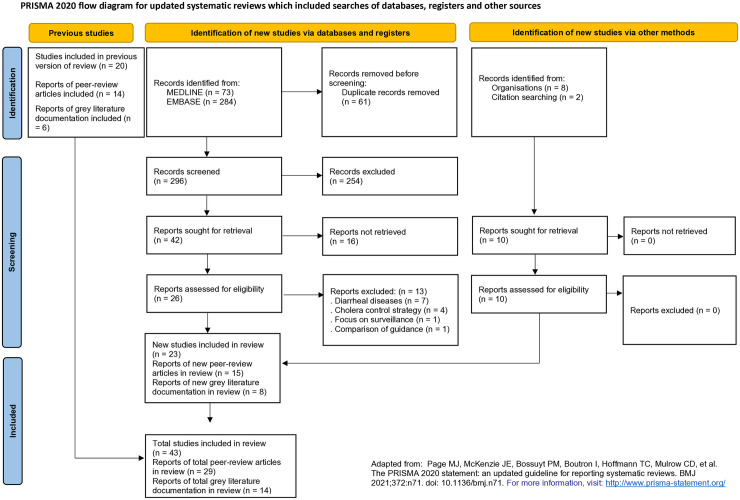
PRISMA flow diagram summarizing the stages of the narrative review process.

To provide transparency and clarity in documenting the selection process, a PRISMA flow diagram was utilized (Fig 1).

### Key informant interviews

The objective of the KIIs was to describe the implementation of CATIs and CATI-like mechanisms in the five countries of DRC, Mozambique, Nigeria, Ethiopia, and Somalia including their programmatic implementation, limitations, challenges, opportunities, and recommendations. We purposively selected researchers, advisors, and implementers experienced in CATIs in those countries.

The countries were selected based on: their cholera burden (with Nigeria and the DRC being two major hotspots in Africa) [28,29]; their experience with original delivery mechanisms (DRC); and, in the case of Somalia (2023), Mozambique (2018), and Ethiopia (2023), the recent implementation of CATI and limited existing documentation. In the DRC, the Ministry of Health [20], Veolia Foundation, London School of Hygiene & Tropical Medicine (LSHTM) [22], Centers for Disease Control and Prevention (CDC) [30], MSF [31] and JHU [24] have conducted studies on cholera targeted interventions. This country has extensive experience with CATIs and CATI-like mechanisms across various contexts, including those marked by endemic cholera, conflict, and remote locations. CATIs have been implemented in Nigeria’s North-East Zone by ACF and SI since 2021, and were evaluated by Johns Hopkins University [18,26]. They were also implemented in 2023 and 2024 by a consortium of national non-governmental organizations (NGOs) led by Goal Prime. The NE zone in Nigeria is characterized by population displacement and hard-to-reach areas due to insecurity and flooding. To our knowledge, and despite frequent cholera outbreaks, CATIs have not been formally evaluated in Mozambique and Somalia. In 2023, the Ministry of Health in Ethiopia, with the technical support of MSF, guided and documented the first known CATI response in the country [32].

Key informants were identified through a combination of a narrative review and recommendations from the GTFCC WASH and Surveillance Working Group leads, particularly for countries where CATI implementation is not well documented. Subsequently, a snowball sampling approach within each country further expanded the network.

We developed a semi-structured interview guide based on the narrative review to collect data (S2 File). Itcovered topics such as experience in CATI, description of CATI delivery, implementation challenges, adaptations, documentation, and referrals to additional interviewees.

### Data analysis

We extracted data from the narrative review into an Excel-based Data Extraction Form (DEF), capturing publication details, study characteristics, interventions, outcomes, and summaries. We then conducted a thematic analysis using the DEF, focusing on contextual settings, challenges during CATI implementation, deployment strategies, and related limitations, opportunities, and improvements. The first author carried out interviews coding and interpretation and discussed and interpreted the findings with the wider team. Data from interviews were analyzed using the qualitative data analysis software NVIVO 14 (Denver, Colorado, USA). Themes identified during the narrative review informed the development of the interview guide.

**Objective 1 -** We established a comparative table, detailing each CATI or CATI-like experience by delivery mechanism, country, and intervention date with information on implementer(s), components, related contexts, implementation challenges, and adaptations. We included CATI and CATI-like experiences that were either documented in peer-reviewed articles or in gray materials or described through interviews. We qualified the experiences by (i) type of WASH intervention (e.g., household disinfection, hygiene promotion, point-of-use water treatment, soap distribution, emergency water supply or repairs, water quality intervention, and/or water quality monitoring), (ii) type of health intervention (e.g., selective chemoprophylaxis, oral cholera vaccine), (iii) type of implementer (e.g., government, non-governmental organizations, Red Cross), and (iv) contextual factors (e.g., territorial context, special conditions, cholera setting, socio-cultural aspects). Contextual descriptors were extracted directly from the original studies or reported by key informants. These terms were included only when explicitly referenced in the source material.

**Objective 2** - We applied deductive coding to analyze the qualitative data, using a framework of implementation challenges derived from the thematic analysis of the narrative review. We then refined these codes by a second-order interpretive coding. Using Coggle (Oxford, United Kingdom), we structured these challenges into a mind map, organizing them into categories and subcategories while incorporating contextual features and their relationships. Each challenge category was qualified as context-specific and/or recurrent or less common. Context-specific challenges were identified based on the links between contextual features and the reported challenges in the mind map. To assess frequency, we used “challenge counts” from both the narrative review and the KIIs. Challenge counts from the narrative review (n) indicate the number of CATI and CATI-like experiences in which a given challenge was reported. Challenge counts from the KIIs reflect the number of respondents who mentioned a given challenge. Frequencies were expressed as percentages across the 18 CATI and CATI-like experiences in which implementation challenges were documented, and across the 15 interviewees. A challenge category was considered ‘recurrent’ if it appeared in at least 25% of the 18 published interventions and was mentioned by at least half of the respondents. It was classified as ‘less common’ if it appeared in fewer than 25% of published cases or was mentioned by less than half of respondents. We synthesized implementation challenges by category, drawing on findings from both the narrative review and the interviews. We also contrasted the challenges identified during the KII with those reported in the literature.

**Objective 3** - Due to the limited number of adaptations made to intervention delivery, a similar classification method to the one used for implementation challenges was not feasible. Instead, the adaptations were grouped into two categories that emerged from the data: the first related to the different CATI-like mechanism strategies, while the second focused on adaptations made to enhance intervention delivery across all strategies. For the second type, adaptations were identified from the narrative review and during interviews, and were synthesized.

**Objective 4** - We summarized contextual features, implementation challenges, adaptations made to intervention delivery, and expected effectiveness improvements in an implementation framework. We defined improvement in effectiveness as response timeliness, expanding coverage, increasing acceptance and uptake, and improving cost-efficiency based on previous reviews [8,14]. We then qualitatively assessed the expected gains in these areas following adaptations.

## Results

We included a total of 29 peer-reviewed articles and 14 gray literature documents after screening from the narrative review (Fig 1; S2 Table). The initial search in MEDLINE and EMBASE yielded 73 and 284 results, respectively. After removing 61 duplicates, 254 records were excluded during title and abstract screening, and 16 were not accessible. Of the 26 reports reviewed in full, 13 did not meet inclusion criteria. Thirteen new peer-reviewed articles were included, along with 14 records from previous reviews by Sikder et al. and Ratnayake et al., and two additional articles identified through citation searching. The grey literature comprised six documents from earlier systematic reviews and eight after the reviews were published.

Additionally, we conducted 15 KIIs with 18 respondents from DRC (n = 4), Mozambique (n = 7), North-East Zone in Nigeria (n = 2), Ethiopia (n = 2), and Somalia (n = 3). There were three group interviews, each involving two participants, conducted in DRC (n = 1), Mozambique (n = 1), and Somalia (n = 1). The respondents included implementers (n = 10), advisors (n = 7), and researcher (n = 1), and were affiliated with national NGOs (n = 6), international NGOs (n = 3), United Nations organizations (n = 5), government entities (n = 2), and one research center (n = 1).

### CATI and CATI-like experiences

#### Description.

A total of 27 distinct CATI or CATI-like experiences were identified through the narrative review and KIIs, and categorized by delivery mechanism, country, and intervention date (Tables 1 and 2). These interventions spanned from 2004 to 2024 and covered 15 countries. The majority of the countries were located in Africa (11/15), including Cameroon, DRC, Ethiopia, Guinea, Mozambique, Nigeria, Sierra Leone, Somalia, South Sudan, Uganda, and Zimbabwe. The other countries were in Southeast Asia (Bangladesh and Nepal), the Caribbean (Haiti), and the Middle East (Yemen).

**Table 1:** Description of CATI and CATI-like documented responses. CATI intervention.

		Type 1: Case-Area Targeted Intervention (CATI) n = 19																		
**Description**	**Country**	Cameroon		Sierra-Leone	Haiti		Haiti, Democratic Republic of Congo	Uganda	South-Sudan	Nepal	Yemen		Zimbabwe	Democratic Republic of Congo			Nigeria	Mozambique	Somalia	Ethiopia
	**Intervention location and date**	Douala (2004)	Kribi (2020)	Freetown (2012)	National (2013-2017)	Centre (2015-2017)	Unknown	Kampala, Katana (2015–2016)	Juba (2015)	Kathmandu Valley (2016)	National (2016-2018)	Hodeidah (2016-2017)	Harare (2018)	North Kivu (2017)	North and South Kivu, Tanganika (2019-2023)	Nort and South Kivu, Kasaï-Oriental, Haut Katanga (2022-2023)	North East (2021)	National (2022-2024)	Belet Hawa, Jubaland (2023)	Somali (2023-2024)
	**Reference**	Noeske, 2006; Guévart, 2007	Ouamba, 2023	Dunoyer, 2013	Rebaudet, 2019; Ramos, 2019; Spiegel, 2021	Michel, 2019	Gallandat, 2020	Bwire, 2021	Parker, 2017	Roskosky, 2019	Spiegel, 2021; Ramos 2019; UNICEF 2018	Altmann, 2018	UNICEF, 2018: Spiegel, 2021	Cardon, 2018; Spiegel, 2021	Endress, 2023; Spiegel 2021	Finger, 2024 KII 07	OKeeffe, 2024; Kaur, 2023	UNICEF, 2024 UNICEF ESARO, 2023 KII 01-02-08-09-10	UNICEF ESARO, 2023 KII 03–05	MSF 2024 KII 06–15
	**Implementer**	Unknown	MoH, MSF	ACF	MOH, WASH NGOs (UNICEF support)	MOH, WASH NGOs (UNICEF support)	MOH, WASH NGOs	MoH	MoH, South Sudanese Red Cross, Oxfam, MSF	MoH	Ministry of Water (UNICEF Support)	ACF	Municipality of Harare, OXFAM, GOAL	SI	Red Cross (UNICEF support)	MSF (Epicentre support)	SI and ACF	MoH, WASH NGOs (UNICEF support)	NCA, Alight (UNICEF support)	MoH, MSF
	**Component**	WASH; ACP	WASH; ACP; OCV	WASH	WASH; ACP	WASH; ACP	WASH; ACP	WASH; ACP	WASH; OCV	WASH	WASH	WASH	WASH	WASH	WASH	WASH; ACP; OCV	WASH	WASH	WASH	WASH; ACP; OCV
**Context**	**Territorial context**	Urban; Densely populated; Slum; Poor WASH conditions	Rural; Urban; Prison	Urban	Rural; Urban; Remote location	Rural; Urban	Urban; Semi-Urban; Densely populated; Camps	Urban; Slum; Poor WASH conditions	Urban; Peri-urban; Densely populated; Slum; Camps; Prison	Urban	Rural; Urban; Poor WASH conditions	Urban	Urban; Poor WASH conditions	Rural; Poor WASH conditions	Rural; Urban; Densely populated; Remote location	Rural; Urban; Peri-urban; Densely populated; Mining site; Remote location; Poor WASH conditions	Rural; Urban; Peri-urban; Densely populated; Rehabilitation center	Rural; Urban; Camps; Remote location; Poor WASH conditions	Urban; Densely populated; Camp; Poor WASH conditions	Urban; Densely populated; Poor WASH conditions
	**Special conditions**				Inaccessible during rainy season		Conflict setting			Natural disaster recovery	Conflict setting	Conflict setting		Conflict setting		Conflict setting	Conflict setting	Conflict setting; Natural disater recovery	Conflict setting; Natural disater recovery	
	**Cholera setting**	Epidemic	Tail	Epidemic	Onset; Peak; Tail	Onset; Peak; Tail	Endemic; Epidemic			Endemic; Onset; Peak	After the peak, Tail	Peak, Tail	Epidemic; Tail	Epidemic; After the peak; Tail	Endemic	Endemic; Epidemic; Onset	Peak; Tail	Onset; Peak; Tail	Peak; Tail	After the peak; Tail
	**Socio-cultural aspect**				Stigma		Mistrust; Stigma						Mistrust	Mistrust	Mistrust		Mistrust	Mistrust; Cultural beliefs	Cultural beliefs	

OCV, oral cholera vaccine; ACP, antibiotic chemoprophylaxis.

**Table 2:** Description of CATI and CATI-like documented responses. CATI-like intervention.

		Type 2: Pre-CATI n = 1	Type 3: Case-cluster intervention n = 2		Type 4: Health-facility-based intervention n = 5				
		Pre-CATI	Cluster	Cluster Grid	Cholera Hospital-Based Intervention (CHOB-7), (PICHA-7)		Household Disinfection Kit (HDK) Distribution		Hygiene Kit Distribution (HKD)
**Description**	**Country**	Democratic Republic of Congo	Guinea	Democratic Republic of Congo	Bangladesh	Democratic Republic of Congo	Haiti		Democratic Republic of Congo
	**Intervention location and date**	Eastern provinces (2021-2024)	Conakry (2012)	Kimpese, Kinshasa, and Mbujimayi (2017-2018)	Dhaka (2014–2015) (2019–2021)	South Kivu (2020-2021)	Port au Prince (2010-2011)	Artibonite (2019-2020)	Kasaï-Oriental (2018)
	**Reference**	KII 11-12-13	Dunoyer, 2013	Bompangue, 2020; Spiegel, 2021; KII 12–13	George, 2016; George, 2016; Saif-Ur-Rahman, 2016; Zohura, 2022; George, 2022	Bisimwa, 2022	Gartley, 2013	Heylen, 2022	D’Mello-Guyett, 2020; D’Mello-Guyett, 2021; Spiegel, 2021
	**Implementer**	Congolese Red Cross (UNICEF support)	ACF, Guinean Red Cross	MoH (WHO support)	MoH	MoH	MSF	Clear Water Haiti	MSF
	**Component**	WASH	WASH	WASH; ACP	WASH	WASH	WASH	WASH	WASH
**Context**	**Territorial context**	Rural; Remote location	Urban, Densely populated, Poor WASH conditions	Urban; Densely populated;	Urban; Slum	Urban; Slum; Poor WASH conditions	Urban; Poor WASH conditions	Urban	Remote location; Poor WASH conditions
	**Special conditions**	Conflict setting					Natural disaster recovery	Conflict setting	Confict setting
	**Cholera setting**	Onset	Epidemic; Onset; Peak and Tail	Peak; Tail	Endemic		Epidemic		Tail
	**Socio-cultural aspect**								Stigma

OCV, oral cholera vaccine; ACP, antibiotic chemoprophylaxis

Around half of the CATI and CATI-like experiences were implemented by a single actor (15/27; 56%), while several involved multiple actors (11/27; 41%), and in one case, the implementation arrangement was unknown (1/27; 3.7%). Government counterparts co-led about half of the responses (15/27; 56%), primarily through the Ministry of Health (14/15; 93%), with one led by the Ministry of Water. In most cases (17/27; 63%), an NGO was involved in implementation. The Red Cross was identified as an implementing actor in four experiences (4/27; 15%). Lastly, large-scale CATI responses spanning provinces, departments or districts were implemented in Haiti, Yemen, Mozambique, and DRC and were supported by UNICEF [33,34,30].

The majority of responses to cholera outbreaks included only WASH interventions (17/27; 63%), while approximately one-third were multisectoral, incorporating both WASH and health components (10/27; 37%). None of the experiences included health interventions alone. All CATI and CATI-like experiences included one or more WASH components: household disinfection (17/27; 63%), hygiene promotion (27/27; 100%), point-of-use water treatment (POUWT) (22/27; 81%), soap distribution (23/27; 85%), emergency water supply or repairs (5/27; 19%), water quality intervention (11/27; 41%), and/or water quality monitoring (11/27; 27%). Selective antibiotic prophylaxis was administered in about one-third of experiences (9/27; 33%), in Cameroon (Douala and Kribi) [15,27], Haiti (National and Centre department) [16,17], Uganda [35], DRC [7,20], and Ethiopia [32]. OCV was administered by MSF and partners in few CATI experiences (4/27; 15%), in Kribi (Cameroon) [27], Juba (South Sudan) [36], DRC [7], and the Somali Region (Ethiopia) [32], using doses left over from previous campaigns or purchased directly from the manufacturer (for the study in DRC).

CATI and CATI-like experiences were conducted in urban areas (15/27; 55%), rural areas (3/27; 11%), and mixed urban-rural settings (8/27; 30%), while the setting was unspecified in few (1/27; 4%) cases. Key contextual features included poor WASH conditions (12/27; 44%), conflict settings (11/27; 41%), densely populated areas (9/27; 33%), remote locations (6/27; 22%), slums (5/27; 18,5%) and camps (4/27; 15%). Interventions were documented in both epidemic (8/27; 30%) and endemic (5/27; 19%) settings, while the outbreak setting was not mentioned in approximately half of cases (14/27; 52%). Community mistrust was noted in about one-fifth of instances (22%; 6/27). Tables 1 and 2 shows the different documented CATI and CATI-like experiences by delivery mechanism, country, and intervention date, between 2004 and 2024.

#### Definition of CATI and CATI-like mechanisms.

**Fig 2:**
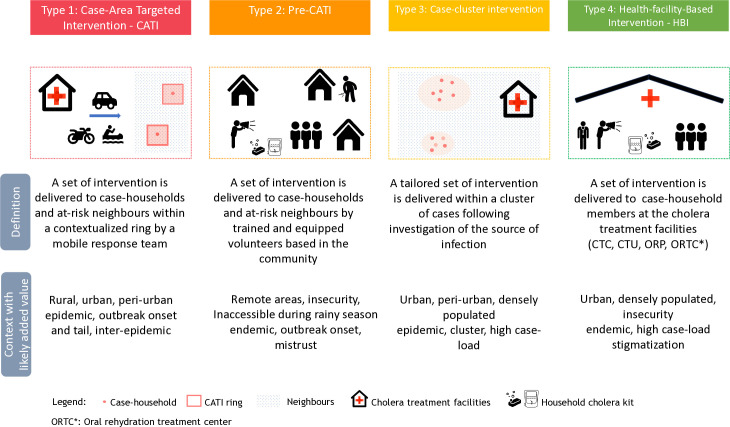
Schematic representation of CATI and CATI-like delivery mechanisms. The figure was created using Wikipedia Commons (https://commons.wikimedia.org/wiki/Accueil).

We identified four types of delivery mechanisms, including CATI and three types of CATI-like mechanisms (pre-CATI, case-cluster, and health-facility based interventions (HBI)) (Fig 2). CATI was documented in 19 experiences, pre-CATI in one, case-cluster in two, and HBIs in five.

CATIs are defined as a multi-sectoral package of interventions delivered promptly after case presentation to case households and at-risk neighbors within a defined ring through mobile response teams (Table 1). This approach accounted for 70% (19/27) of all experiences and was the most extensively documented form of delivery mechanism [15–18,26,27,30–46]. Overall, 89% (17/19) of CATI experiences were conducted in urban settings, while nearly half (9/19; 47%) were in rural settings. Interventions occurred more frequently during the peak (7/19; 37%) and tail (10/19; 53%) phases than at the onset (4/19; 21%) of outbreaks.

Pre-CATIs are designed to occur before the mobile CATI team reaches cholera affected areas. Volunteers and community healthcare workers are trained to deliver CATI interventions upon case detection. This delivery mechanism was developed and scaled up by the Congolese Red Cross, with UNICEF support, in 2021 across three provinces in DRC (Table 2) (from KII). The documented Pre-CATI was carried out in rural, remote, and conflict-affected areas at the onset of the outbreak.

The case-cluster mechanism involves delivering a tailored set of interventions within a cluster of cases, based on georeferencing of case households and investigating potential sources of infection, with a focus on emergency water supply, and water quality intervention and monitoring at community-level (Table 2). The duration of the intervention is typically longer than in the CATI response, lasting up to 14 days in the grid strategy. Case-cluster mechanisms were documented in two instances: Conakry, Guinea (2012) by Action Against Hunger [19] and Kinshasa, DRC (2017) by the Ministry of Health with the support from the World Health Organization [20]. In Conakry, the case-cluster mechanisms included CATI activities such as daily georeferencing, home-disinfection, distribution of cholera kits, and hygiene promotion for affected households. Additionally, it involved identification and implementation of tailored interventions such as repairing a portion of the water distribution network and conducting tailored hygiene promotion for high-risk groups in persistent clusters [19]. In Kinshasa, a grid strategy consisted of mapping cases, marking 500-meter clusters, and conducting 14-day WASH interventions by community volunteers and selective chemoprophylaxis in each grid [20,30]. Both case-cluster experiences were conducted in urban and densely populated settings, during the peak and tail of a cholera outbreak [19,20].

HBIs were documented in five responses in Haiti [21,47], Bangladesh [23,25,48–50], and DRC [22,24,51] (Table 2). Subtype 1 involved distribution of different types of kits (e.g., hygiene kits, disinfection kits) to caregivers in MSF Cholera Treatment Centers in Haiti (2010–2011) [21] and Kasai Oriental, DRC (2017) [22,51]. Subtype 2 included Cholera-Hospital-Based-Intervention-for-7-Days (CHOBI-7) [23,48] and the Preventative-Intervention-for-Cholera-for-7-Days (PICHA-7) [24] programs, which have been piloted in Bangladesh and the DRC by JHU since 2014. These HBIs aim to reduce cholera transmission during the seven-day high-risk period for patients and their households by promoting safe WASH practices, including handwashing, water treatment and safe storage, through formative research for program design and in-person visits, and/or bi-weekly mobile messages for program delivery [24,50]. The majority of HBI experiences (4/5; 80%) were conducted in urban settings [21,23,24,47,48], with some in slum areas in Bangladesh and DRC [23,24,48]. Conflict settings were reported in two HBIs in Haiti and DRC [22,30,47,52]. Fig 2 shows the different types of approaches to provide WASH and health services to cholera-affected households and their neighbors.

### Implementation challenges

We identified ten categories and 50 sub-categories of implementation challenges (S1 Fig; Table 3). Within the ten categories, four were considered context-specific, including access to case-households, delayed response activation, lack of resources, and low uptake and refusal (S1 Fig). Five categories were identified as recurrent, including lack of resources to initiate, maintain, or scale-up the response (61% of KII, 11 out of 18 published interventions), delays in response activation (44%, 8/18), limited skills and knowledge (44%, 8/ 18), difficult access to case-households (33%, 6/18), and overwhelmed response capacity (33%, 6/18) (Table 3). Less common challenges included low WASH uptake (22%, 4/18), low acceptability and refusal (22%, 4/18), weak coordination (22%, 4/18), poor reporting and monitoring (17%, 3/18), and sustainability issues (11%, 2/18). Although sustainability issues and low acceptance/refusal were less documented in the literature, they were frequently discussed during interviews. Since there were no differences in implementation challenges across delivery mechanisms and less evidence for CATI-like mechanisms, we present the consolidated data in S1 Fig In this, we highlight the different categories (rectangular box), and sub-categories (oval box) of implementation challenges, and contextual features (notched rectangular box), and their relationships.

**Table 3:** Summary of implementation challenges, by category and sub-category, for CATI and CATI-like approaches.

Implementation challenges	KII sources (n)	KII sources (%)	NR responses (n)	NR responses (%)
**1. Delay in response activation**	**10**	**67%**	**8**	**44%**
Contractual arrangements delay	5	33%	0	0%
Response funding delay	4	27%	0	0%
Inadequate response trigger	4	27%	3	17%
Political and institutional barrier	4	27%	2	11%
Lack of in-country competency	1	7%	2	11%
Delays in operational deployment	3	20%	1	6%
Late hygiene kit access	0	0%	3	17%
Travel time before the response	1	7%	1	6%
**2. Challenging access to case-households**	**7**	**47%**	**6**	**33%**
Issues in locating case-households	4	27%	3	17%
Insecurity	3	20%	3	17%
Poor road condition	2	13%	0	0%
Remote location	1	7%	1	6%
Weathers constraints	0	0%	2	11%
Traffic in dense urban areas	0	0%	1	6%
**3. Lack of resources**	**12**	**80%**	**11**	**61%**
Lack of funding	8	53%	3	17%
Limited human resources	4	27%	5	28%
Resource-intensive	8	53%	1	6%
Restricted OCV access	1	7%	3	17%
Supply stock out	7	47%	3	17%
**4. Overwhelmed response capacity**	**6**	**40%**	**6**	**33%**
High case load	4	27%	6	33%
Low specificity of cases targeted	3	20%	1	6%
Difficulty to cover large-ring size	0	0%	2	11%
**5. Lack of skills and knowledge**	**10**	**67%**	**8**	**44%**
Low knowledge and experience level	8	53%	4	22%
Poor Interaction with families	1	7%	0	0%
Research gap	1	7%	0	0%
Lack of standardization for delivery	0	0%	5	28%
Unspecific and incomplete strategy	7	47%	1	6%
Weak surveillance	4	27%	1	6%
6. Low WASH uptake	2	13%	4	22%
Water scarcity	1	7%	4	22%
No qualitative kit demonstration	0	0%	1	6%
Diversion of intended use	1	7%	1	6%
Intervention not adapted	1	7%	1	6%
7. Low acceptance and refusal	9	60%	4	22%
Cultural or religious beliefs and practices	3	20%	2	11%
Excessive chlorination	1	7%	0	0%
Kits stock out or inadequate content	2	13%	1	6%
Misinformation	2	13%	0	0%
Mistrust	2	13%	3	17%
Stigmatization	4	27%	1	6%
8. Weak coordination	3	20%	4	22%
Data sharing issues	1	7%	2	11%
Health rapid response teams and CATI duplication	1	7%	2	11%
Lack of shared responsibility across sectors	2	13%	0	0%
No medical activities (ACP, OCV)	0	0	1	6%
9. Poor reporting and monitoring	4	27%	3	17%
Kobo not filled consistently	1	7%	0	0%
Inadequate reporting form	1	7%	0	0%
No use of monitoring to adapt the response	1	7%	1	6%
Other	3	20%	2	11%
10. Sustainability issues	6	40%	2	11%
Issue with payment of volunteers	3	20%	0	0%
Lack of institutional memory	1	7%	0	0%
Long-term engagement issues	2	13%	0	0%
Low Ministry of Health appropriation	1	7%	2	11%

The context-specific challenge categories are shaded in grey, while the recurrent ones are in bold.

NR, narrative review.

Each of the ten implementation challenge categories are further described below.

*Delays in response activation*. Political, administrative, logistical, and procedural constraints caused delays. Respondents mentioned that there were delays in response in the North-East Zone in Nigeria and in Mozambique due to political challenges in declaring cholera outbreaks. Additionally, unclear CATI activation triggers (e.g., whether to act once an outbreak is declared, after one confirmed case, or based on RDT results) and differences in disease control objectives (e.g., slowing down of the outbreak, stopping the transmission) between donors, governments, and implementers created coordination challenges, further complicating timely response efforts (from KII). In Mozambique [34] (from KII), Somalia [46] (from KII), and the DRC (from KII), UNICEF-led efforts faced delays due to funding or contractual issues, with response activation one to two months after the outbreak began. Logistical challenges also contributed to delays. Respondents from the DRC, Mozambique, and Somalia noted that a minimum two-week setup period was required for training personnel and organizing responses, particularly in remote or newly affected areas. Furthermore, some response efforts were delayed by procedural constraints that implied mandatory laboratory culture confirmation for each case in Nepal and for each new affected province in an MSF-led study in DRC (from KII). In Nepal, the average time between patient admission and CATI implementation was 3.9 days, with culture results taking approximately 3 days [39]. KIIs helped clarify details about the delays in response activation, especially those linked to funding delays, internal contractual issues, inadequate response triggers and political sensitivities.

*“We had to establish the proposal, the contracts, and when the funds reach to the organizations, it took a lot of time to have the resources.” (Mozambique)*

*“The second point is the delay of the health authorities to declare the cholera outbreak, and you cannot start the activities if they didn’t declare the outbreak.” (Mozambique)*

*“There’s a bit of confusion on that [CATI activation]. Donors, partners and the government don’t always have the same threshold.” (Nigeria)*

*Challenges to access case households*. This was primarily due to difficulties in locating patient homes, often caused by incomplete or missing addresses [26,30,39,53]. These challenges arose either from insufficient information recorded at cholera treatment facilities or from patients’ reluctance to disclose their location. Difficulties in reaching case homes were also highly context-specific, influenced by factors such as insecurity [18,53], restricted access during the rainy season [16,44], remote locations [44], and travel time in densely populated urban areas [18].

*“We have lots of cases where patients don’t want to give their exact address.” (Mozambique)*

*Lack of resources.* This included inadequate funding [16,18,44,53], insufficient human resources [34,36,44,46], stockouts of supplies [18,26,27], and limited access to OCV [27,34,36,39]. Multiple crises triggered limited human resources to respond to cholera outbreaks in Mozambique and Malawi [34,45]. Restricted access to vaccines was due to shortages in the Global OCV stockpile [34,36] and the OCV application mechanism, which does not have a policy for CATI delivery [27,36]. In half of the interviews, respondents cited lack of funding and supply stockouts, underscoring the critical importance of these challenges. Additionally, the resource-intensive nature of CATI was frequently discussed, despite being rarely documented in the literature.

*“[The challenges of CATI are that] they are late and insufficient in terms of resources.” (Multi-countries)*

*Overwhelmed response capacity*. This occurred during high caseloads in multiple settings [17,18,26,32,33,39,42], exacerbated by over-reporting of suspected cholera cases due to the low detection capacity [44] and the low case definition specificity [40] in Yemen, Somalia (from KII), and Mozambique (from KII). In the context of high caseloads, additional challenges were noted, including difficulties in covering large ring sizes in Ethiopia [32] (from KII) and Nigeria [26], followed by supply stockouts reported by respondents (from KII).

*“The main challenge we had with the CATI response is the nature of the cases that were received at the Cholera Treatment Unit. We felt there was not a proper case definition.”* (Somalia)

*Lack of skills and knowledge*. In Yemen, a lack of expertise and preparedness resulted in a generic WASH response, with untargeted activities aimed at improving water access and solid waste management [38,40]. Response plans were updated only during the second wave and shortly after the peak, when UNICEF and WHO deployed cholera specialists to develop a cholera-specific control strategy, including CATIs [38]. Weak surveillance capacity in Mozambique [34] (from KII) and Somalia (from KII) hindered case detection and data management. In countries that had recently implemented CATIs for the first time such as Somalia and Ethiopia, finding skilled partners in cholera targeted interventions was challenging (from KII). Moreover, a lack of standardization when multiple implementers were involved in UNICEF-supported responses was highlighted [16,44,45,49]. Eventually, in Yemen [33], Mozambique (from KII) and Somalia (from KII), the absence of an evidence-based, integrated strategy created a disconnect between CATIs and other control activities with geolocation of case-households and case investigation being underutilized. Concerns about disengagement from cholera control interventions beyond CATIs such as Risk Communication and Community Engagement (RCCE), emergency water supply, and water quality intervention and monitoring were raised in six interviews (from KII). In all five countries where key informant interviews were conducted, a lack of skills and knowledge among implementers was identified as a challenge in CATI implementation. Several respondents also highlighted the nonspecific and incomplete nature of the cholera response, emphasizing the risk of diverting resources and efforts exclusively toward CATI. This concern was raised in nearly half of the interviews but was rarely discussed in the peer-reviewed or gray literature.

*“This part of investigating, analyzing and understanding epidemic dynamics, which is very weak. We have a lot of data, but nobody’s looking at it.” (Mozambique)*

*“They [stakeholders] were looking at us like kind of magic bullet. Now [that] CATI is implemented, everything will be okay. So, they kind of stopped other interventions.*” *(Ethiopia)*

*“I think there are mandatory actions to any [cholera] response and that allow to improve the impact of CATI is going to be the chlorination of everything that can be chlorinated”. (Multi-countries)*

*Low WASH uptake.* This was reported during MSF-led CATI responses in the DRC [31] (from KII) and Ethiopia [32] (from KII), as well as during an MSF-led HBI intervention in the DRC [22]. It was also noted in formative research for the design of CHOBI-7 in Bangladesh [50] and PICHA-7 in the DRC [24]. Contributing factors included households lacking sufficient water for handwashing, repurposing supplies for other needs (e.g., washing clothes), and concerns about over-chlorination and taste.

*“There was no water for hand-washing. Even for drinking, there was no water”. (Ethiopia)*

*“We didn’t check any kind of free residual chlorine […]. Some people, they were kind of like complaining [of the taste of the water]”. (Ethiopia)”*

*Low acceptance and refusal*. Low acceptance of interventions was driven by community mistrust [26,43,44,53] and [44]. Refusals were reported in Nigeria and DRC when no male household members were present [26,43], in Nigeria [26] and Somalia (from KII) due to lack of supplies, and in Mozambique (from KII) and DRC [53] where fear of stigmatization arose from the visibility of household spraying teams. Low acceptance and refusal of CATI were discussed in two-thirds of the interviews and reported across all five countries, although this issue was less frequently reported in the narrative review.

*“They believe this is brought from […] foreigners or someone who come from outside who brought cholera with the [chlorine] powder”. (Mozambique)*

*“There are some houses that the religion or their culture do not permit for men to come into the compound […] and most of the chlorinators were men”. (Nigeria)*

*Weak coordination*. A lack of agreement with surveillance teams led to delays in CATI teams accessing patient's location information in various settings [19,30,34]. Similarly, insufficient coordination between the Health and WASH sectors led to the creation of parallel “Health” and “WASH” rapid response teams in Haiti, Yemen [33,38], and Nigeria. Additionally, in nearly all WASH-led CATIs (86%, 12/14), there was an absence of medical interventions, such as chemoprophylaxis and OCV [30].

*“If we don’t have access to data every day on cases and their addresses, we can’t respond. That’s an insurmountable first limit.” (Multi-country)*

*Poor reporting and monitoring*. Insufficient skills and constrained human resources were identified as major barriers to effective CATI reporting, monitoring, and subsequent data analysis (from KIIs). Partners’ limited access to the Kobo database, a digital tool commonly used to report CATI activities and monitor implementation, made it difficult to use reporting or monitoring results to guide other control activities or adjust the ongoing response. This challenge was not well documented in the literature but was mentioned in nearly one-third of interviews (27%).

*“The partners barely have access to the Kobo thing. They don’t really have the capacity to analyse the results. I end up with a huge database and I don’t really know what to do with it.” (Mozambique)*

*Sustainability issues*. Lack of sustainability was observed in multiple responses [30,44], which faced challenges such as high operational costs (from KII) and low government ownership [30]. Additionally, the Ministry of Health in Mozambique (in certain provinces) and Ethiopia expressed concerns that paying incentives to volunteers would disrupt the community health network approach (from KII). This was frequently discussed during interviews but was rarely mentioned in the literature.

*“I feel that they see CATI as something that UNICEF and other partners are bringing to them [Ministry of health]. It seems like something that’s coming from outside. It’s not their work.” (Mozambique)*

*“We are living in a low-income country. We cannot just sustain such incentives […] at a government level. […] Providing incentive for community […] may disturb even the community health systems.”*

### Adaptive strategies

The results are presented in two parts: the first one describes the CATI-like mechanisms that emerged in response to challenging contexts, while the second one focuses on adaptations made to enhance intervention delivery.

### CATI-like mechanisms

Limitations associated with CATI implementation included reduced coverage during high caseloads [26], resource-intensive nature of the delivery mode [8], high costs [30,47], and challenges in reaching hard-to-reach areas [47]. In response, CATI-like delivery mechanisms were developed for challenging contexts, including pre-CATI, case-cluster, and HBI.

The pre-CATI approach was developed in DRC in remote and conflict-affected areas to reduce response delays by training and equipping Red Cross volunteers in the community (from KII). When the number of cases exceeds the response capacity of pre-CATI teams, mobile CATI teams provide additional support. However, limitations included the absence of medical activities and restricted mobility outside the headquarters area. The effectiveness of pre-CATIs has yet to be evaluated.

Three respondents described the “CATI light” concept in Mozambique, where mobile teams targeted case-households only (no ring intervention) during high caseloads due to restricted funding and limited number of implementers, paired with neighborhood-level RCCE, hygiene promotion, and distribution of POUWT (from KII).

The case-cluster approach in Guinea and DRC was also developed in the context of high case-load in densely populated urban settings where regular CATI implementation would result in overlapping rings in clusters and duplication of activity, likely limiting the number of cases responded to due to resource constraints [19,20]. The grid approach has been reported as logistically complex to implement and resource-intensive (from KII).

HBIs have shown promise in endemic and insecure environments with remote interventions from the health facility [23,25,52]. This approach involved challenges with delayed kit distribution upon patient discharge in Mozambique (from KII) and MSF-led responses in Haiti [21] and DRC [52] as well as issues with water scarcity in Ethiopia [32] and DRC [24,52]. The CHOBI-7 program has led to a reduction in diarrheal prevalence [23] and sustained WASH behavior change in case-households in an endemic urban slum in Bangladesh [25]. In a hygiene kit distribution project in DRC, household contacts who used kits experienced a 66% reduced incidence [22].

*“In our context […] endemic to cholera […] and of war and conflicts, we have used Pre-CATI teams […] to access the area during the very first notification. We provide them with resources, logistical resources, materials, inputs.” (DRC)*

*“We use CATIs to be able to respond when there were very few cases. But in the event of an increase in the number of cases, we have this approach, this second community-based approach, which is the grid approach.” (DRC)*

### Adaptations for intervention delivery

We identified 10 different areas where adaptations or recommendations were made to improve intervention delivery for CATI and CATI-like mechanisms: early detection, readiness and activation, case prioritization, ring-size adjustments, resource optimization, OCV integration, intervention uptake and acceptance, knowledge and capacity strengthening, regular reporting and monitoring, and enhanced sustainability. Below, we synthesize the results by areas of improvement.

*Early detection*. Linking community-based surveillance networks with CATI teams and training healthcare workers in cholera diagnosis and the use of rapid diagnostic tests (RDTs) was recommended to increase response promptness [39,54].

*“It is better if cases are identified in the community by the surveillance team and are linked to CATI teams for quick response, rather than waiting for patients to come to treatment facility.” (Ethiopia)*

*Readiness and activation*. Several CATI researchers suggested aligning CATI activation triggers with cholera case definitions from the Global Task Force on Cholera Control (GTFCC) in national cholera plans before outbreaks [30,31,54]. In May 2024, the Health and WASH sectors in North-East Nigeria agreed to use the GTFCC’s newly defined probable case definition to deploy CATI teams [55] (from KII). To improve readiness in various settings, recommendations included prepositioning of response supplies [19,30,33,43], securing early funding [20,26,33], collaborating with local authorities [31], and establishing contingency contracts with CATI implementers, especially in UNICEF-funded responses [34,42] (from KII). Promptly sharing or giving access to patient’s details [33] could be facilitated through prearranged data-sharing agreements (from KII), especially when the WASH sector leads the CATI response [55].

*Case prioritization*. In documented experiences, response prioritization involved selecting areas based on various factors, including higher reported incidence, severe dehydration levels, an elevated case-fatality ratio, and a greater number of community deaths. CATI implementers also selected areas outside vaccinated zones in South-Sudan [36] and focused on responding only to cases identified using enriched RDTs in the DRC (from KII). In Yemen, where RDTs were unavailable, the targeting of case clusters —such as 20 cases within three days in a single village—were used to increase specificity [33]. Prioritizing alerts for severe outbreaks in Haiti with a red-yellow-green alert system [16] and equipping healthcare workers with enriched and point-of-care RDTs in other contexts [31,36,39,54] were further recommended to optimize responses.

*“CATI is pretty resource intensive. If you trigger it for simple diarrhea, all the time, you lose resources pretty quickly, anyway.” (DRC)*

*Ring-size adjustments*. CATI ring sizes varied between published recommendations (100–500 meters) [7–9] and real-world responses, as seen in Ethiopia [32] (from KII), North-East Nigeria [26], and MSF-led CATIs in the DRC [31]. Balancing coverage of more case households and neighbors required adjusting ring sizes based on context, considering factors such as population density, resources and geography [26,31,32]. Smaller rings were preferred in urban areas, especially during high case numbers, while rural areas allowed for more flexibility. In Ethiopia, ring sizes grew larger as cases declined [32] (from KII). The inability to delimit a ring in meters was identified as a challenge by CATI implementers [26]. However, the use of mapping technology, such as Mergin (London, United Kingdom), in the MSF-led operational research in the DRC helped the team cover a defined radius and identify overlapping zones (from KII).

*“Since there were many cases coming per day, we had to decrease the number of households we have to include in CATI. After a while, we again increased this number of households.” (Ethiopia)*

*Resource optimization*. During the 2023 cholera outbreak in Adamawa State, Nigeria, resource optimization was achieved by recruiting community volunteers to reduce transport costs and partnering with national NGOs to pool human resources, thereby reducing overhead expenses (from KII). Integrating CATI teams across sectors and pillars helped to minimize duplication and maximize resource efficiency [30]. In Haiti, the collaboration between WASH NGOs and nurses from the Ministry of Health facilitated the administration of targeted antibiotic prophylaxis to case-household members. Distributing cholera kits directly via cholera treatment centers, cholera treatment units, oral rehydration points, or community health workers reduced operational costs, such as car rentals, minimized delays in kit utilization, and improved coverage in hard-to-reach areas [8,21,47,54]. Additionally, mobile health components in the CHOBI-7 program in Bangladesh minimized the need for physical home visits, enhancing operational efficiency [48]. Mobile messages in both the CHOBI-7 and PICHA-7 programs as well as those recommended in the UNICEF-led response in the DRC [44] helped reinforce proper WASH behaviors. Distributing disinfection materials can boost self-efficacy and promote early, repeated home disinfection during the one week high-risk period [47], while evaluating behavior change beyond this period can improve program effectiveness [22]. In Nigeria, the distribution of standard cholera kits to neighboring households within CATI rings was impossible due to resource constraints, prompting discussions on a low-cost cholera kit within UNICEF’s common pipeline (from KII).

*Intervention uptake and acceptance*. Low acceptance and refusal was addressed by engaging community health workers [8], community leaders [32], and local stakeholders to reduce stigma and increase trust [26,34,44]. Community volunteers and leaders integrated into CATI teams played a key role in facilitating access to case households in Red Cross-led responses in the DRC [30] (from KII) and Ministry of Health-led responses in Ethiopia [32] (from KII). Using survey results, formative research in Bangladesh [48] and the DRC [24], and rapid participatory assessments [43] were effective in adjusting responses and identifying high-acceptance kits. Contextualizing WASH interventions based on local norms, preferences [31,52], and building rapport with households can further improve program acceptance.

*“Often, we have teams that are really vertical. They go in, hand over the kit, throw out four messages and leave. An SBC [Social Behavior Change] specialist was really good to empower the teams in this interactive approach.” (multi-countries)*

*Knowledge and capacity strengthening*. This would require deploying experienced cholera specialists to develop and roll out CATI and CATI-like strategies, particularly in contexts with no previous CATIs experience [38]. Formulating clear, context-adapted standard operating procedures that address specific and recurrent challenges, along with training staff [18,26,38] were suggested to ensure standardization across implementers before the cholera season [31,39,44].

*“One CATI experience does not fit all. It must be contextualized and flexible.” (DRC)*

*Regular reporting and monitoring.* Weekly reporting of CATI implementation with standardized indicators in UNICEF-funded CATI response, such as the percentage of reported cases responded to within 24–72 hours [30,34], improved the timeliness and coverage of CATI responses in Haiti [30]. Monitoring, including surveys to adjust intervention design, was used in Haiti’s household disinfection kit project [21] and recommended in Nigeria [26]. The need for qualitative and systematic post intervention monitoring was consistently highlighted as a priority in interviews with UNICEF advisors and implementing partners.

*Enhanced sustainability.* This was achieved in some UNICEF-led responses by integrating CATIs into the national cholera control plan [33,39] and strengthening and supporting existing disease outbreak response systems [18], such as the municipality rapid response teams in Zimbabwe [42] and contact tracing teams in Mozambique (from KII).

*“Instead of systematically trying to create a whole team through an NGO, we can support this existing system and complement it.” (Mozambique)*

## Discussion

We identified four types of CATI and CATI-like mechanisms implemented in 15 countries, across diverse geographical contexts and outbreak settings. Our analysis revealed 10 categories of implementation challenges and 10 areas of adaptations to improve response delivery. The CATI model, in which mobile teams intervene at case households and surrounding rings, was found to be less suitable in remote locations (due to deployment time), conflict zones (due to restricted access), during high caseloads (due to resource demands and response duplication), and sometimes in areas with community mistrust. Considering context-specific CATI-like mechanisms and adapting intervention delivery to address implementation challenges could help improve response effectiveness by reducing intervention delays, increasing case and ring coverage, enhancing acceptance and WASH behaviors, improving cost efficiency, and ultimately decreasing cholera incidence. Pre-CATI can be particularly valuable in remote or conflict-affected areas, where locally integrated Red Cross volunteers or community health workers can respond quickly. The case-cluster approach helps avoid duplication of activities and enables targeted, tailored interventions in persistent case clusters, while used in combination with CATI for case-households outside of these areas. Interventions from cholera treatment facilities provide a cost-effective alternative or a complementary solution to CATIs to reach case households when mobile teams can not access an area, during high caseloads when resources are limited, or in endemic settings with a steady but relatively low number of daily cases. We summarized our findings into two implementation frameworks for CATI and CATI-like mechanisms, detailing objectives, contextual applications, challenges, adaptations, response phases, and anticipated improvements in effectiveness (S2 and S3 Figs).

There is limited knowledge and understanding among practitioners regarding CATI-like mechanisms and how they can be used independently or combined with CATI. For example, integrating CATI with household disinfection kit distribution—in which mobile teams deliver disinfection supplies, such as bleach together with infographics on instructions for use —could enhance case-household self-efficacy and facilitate early and repeated disinfection, rather than relying solely on one-time interventions by CATI teams [47]. Additionally, the mobile health components of the PICHA-7 and CHOBI-7 programs could be used during CATIs or case-cluster interventions to enhance behavior change [24,50]. We and CATI-like mechanisms in overstretched response contexts, particularly when CATI teams are unable to meet their pre-agreed objectives for case coverage or response time. Case-households that cannot be reached by CATI teams can be addressed at health facilities through HBI. This approach is also applicable in situations where resistance, conflict, or stigmatization hinders the access or acceptance of mobile response teams within the community. In densely populated urban areas with high case-loads and overwhelmed response capacity, we recommend to adopt a case-cluster or a grid approach rather than CATI, through georeferencing and tailored interventions at the community and case-household level. Practitioners should be equipped with the skills and knowledge to implement these shifts effectively. Current guidelines primarily focus on CATI delivery, while neglecting CATI-like mechanisms and the critical processes of reviewing implementation challenges and adapting CATI delivery before and during outbreaks. To address this gap, specific guidelines, processes, training materials, and tools should be developed based on the proposed framework (S2 and S3 Figs). These resources would provide practical support for teams struggling to meet performance objectives during a cholera response and could play a valuable role during preparedness and readiness phases. Furthermore, evidence is lacking regarding the key performance indicators and thresholds to use, particularly when to shift or combine delivery mechanisms. Further discussion and consensus with specialists are needed.

Our analysis of CATIs and CATI-like mechanisms revealed that few responses were initiated at the onset of outbreaks, despite the critical importance of timely intervention. Risk modeling studies [7,55] and a quasi-experimental analysis of CATI implementation in Haiti [17] demonstrate that rapid responses can significantly reduce both the number of cases and the duration of an epidemic. These findings underscore the need for readiness efforts in Priority Areas for Multi-sectoral Interventions (PAMIs), as well as the anticipation of challenges and necessary adaptations during CATI implementation. In conflict-affected or hard-to-reach PAMIs, the timeliness of outbreak response can be enhanced by establishing pre-CATI and engaging, training and equipping national NGOs and/or community-based volunteer networks, and/or district-level intersectoral rapid response teams from the government before the cholera season.

Integration of CATI and CATI-like mechanisms into national plans has enhanced government ownership of these strategies in Haiti, the DRC, and North-East Nigeria, where they have been implemented for several years. While the integration between the WASH and Health sectors improved in Haiti from 2010 to 2018 [16], it did not result in a more sustainable response capacity within the Haitian government or deeper integration into the Ministry of Health’s disease outbreak response mechanisms. This can partly be explained by the deterioration of the security context and the emigration of skilled professionals since 2019 [56]. Furthermore, our review found that when WASH partners lead the response, critical surveillance and medical activities—such as case investigation, ACP, and OCV—were less likely to be carried out. Leveraging existing outbreak response systems could provide additional benefits such as sustainability and ownership and enhance the impact of interventions through stronger intersectoral collaboration.

This study provides valuable insights into the implementation of CATIs in under documented countries such as Somalia, Ethiopia and Mozambique. We introduce and document the “CATI light” concept in Mozambique and the pre-CATI mechanism in DRC. We categorized CATI and CATI-like mechanisms during cholera outbreaks and illustrate the potential of those approaches across diverse contexts. As this study covers diverse geographic contexts and outbreak settings across 15 countries, these findings may be broadly applicable.

Some study limitations should be noted. While the narrative review involved a systematic approach, it did not follow the comprehensive scope of a full systematic review. Indeed, screening, quality/bias assessment, and data extraction was carried out by a single reviewer, which may increase the risk of missing key publications unintentionally. Furthermore, this study relied on MEDLINE and EMBASE as the primary databases for the literature review. An initial scoping search, along with findings from two previous reviews conducted by JHU and LSHTM, confirmed that the majority of relevant literature on CATI and CATI-like experiences was indexed in these two databases. However, the exclusion of other databases may have limited the capture of regionally published studies. Restricting the review to only English and French language publications may inadvertently limit the extent of the review, excluding potentially relevant publications in Portuguese, Arabic and other languages. A single interviewer was responsible for KIIs over a short timeframe, thus restricting KIIs to five African countries. As a result, countries with notable experience in CATIs, such as Haiti and Bangladesh, and other countries with gaps in CATI research, such as Comoros, Mayotte, Malawi, Lebanon and Syria were excluded from KIIs. Key informants were identified through a snowball approach. While some informants may have previously been colleagues of the study team, given the niche nature of the topic, the sampling strategy prioritized individuals and organizations directly involved in CATI implementation to ensure the collection of relevant and context-specific insights. Data-sharing issues between surveillance or medical teams and CATI implementers were not frequently reported during interviews, possibly due to recall bias, as access to health data typically improves over time. Ultimately, the narrative review and KII did not capture the interventions conducted by the Ministry of Health’s rapid response team at the district level during cholera outbreaks. Due to limited documentation, future research could explore how these interventions align with or complement CATI and CATI-like mechanisms.

## Conclusions

This study offers a classification of CATI and CATI-like mechanisms, alongside a comprehensive mapping and categorization of implementation challenges and adaptations to intervention delivery. We recommend that the proposed implementation frameworks and associated findings be reviewed and discussed by subject-matter experts at the global level. Such engagement could support broader dissemination and practical uptake by field practitioners, and inform the development of an operational guideline on CATI and CATI-like mechanisms.

Future studies should evaluate the effectiveness of pre-CATI, case-cluster, CHOBI-7 and PICHA-7 approaches during cholera outbreaks. Additionally, assessment of the cost-effectiveness of various delivery mechanisms, whether used independently or in combination, may guide resource allocation. CATI and CATI-like mechanisms primarily aim to reduce household transmission and often address environmental transmission through improved access to safe water, though they rarely encompass all contexts of transmission. Therefore, they should be integrated into a multi-sectoral and comprehensive cholera control strategy, informed by epidemiological data, case investigation findings, socio-cultural drivers, and WASH assessments. This strategy should also promote complementary interventions such as RCCE, emergency water supply, water quality measures, and ongoing monitoring. While CATI is not a magic bullet, it serves as the cornerstone of an evidence-based and targeted response.

## Supporting information

S1 Fig Mind map of implementation challenges for CATI and CATI-like approaches. (TIF)

S2 Fig Implementation framework for CATI delivery mechanisms. The color-coding system is as follows: red for CATI, orange for pre-CATI, yellow for case-cluster, and green for HBI delivery mechanisms. Adaptive strategies are divided into (1) adaptations to the existing delivery mechanism (blue rectangles) and (2) suggestions to shift or combine with other delivery mechanisms (color rectangles). The figure was created using Wikipedia Commons (https://commons.wikimedia.org/wiki/Accueil).
(TIF)

S3 Fig Implementation framework for CATI-like delivery mechanisms. The color-coding system is as follows: red for CATI, orange for pre-CATI, yellow for case-cluster, and green for HBI delivery mechanisms. Adaptive strategies are divided into (1) adaptations to the existing delivery mechanism (blue rectangles) and (2) suggestions to shift or combine with other delivery mechanisms (color rectangles).
(TIF)

S1 Table Sources for searching gray literature resources. (XLSX)

S2 Table List of peer-review articles included in the study. (XLSX)

S1 File Search rings and results for the narrative review. (DOCX)

S2 File Semi-structured interview guide. (DOCX)
