# Supplementary figures and images for: Optimizing the implementation of case-area targeted interventions during cholera outbreaks with context-specific delivery mechanisms
Source: PLoS Negl Trop Dis. 2025 Sep 23;19(9):e0013534. doi: 10.1371/journal.pntd.0013534 (PMC12456772; doi:10.1371/journal.pntd.0013534)

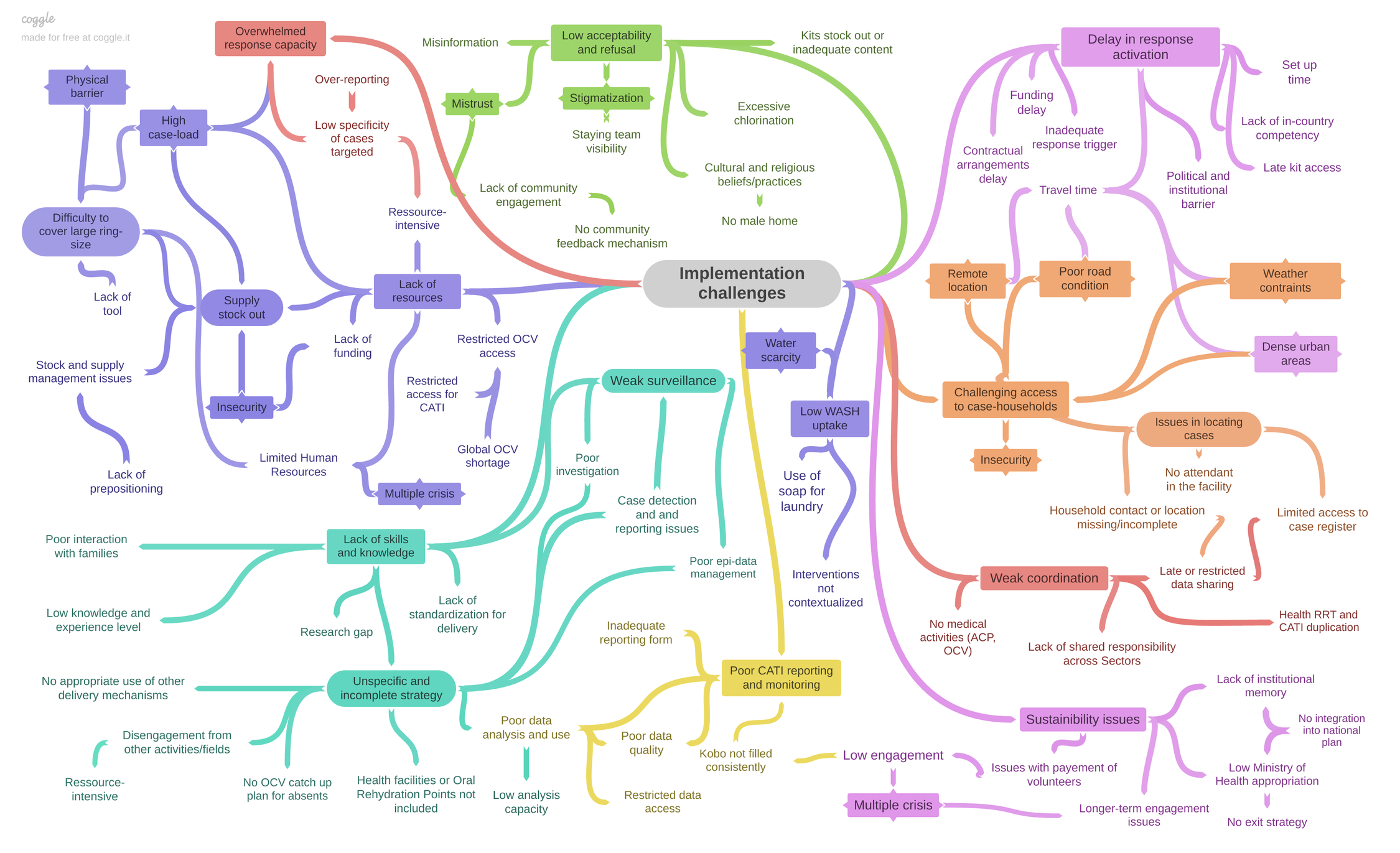

Supplement: S1 Fig — (TIF) [file pntd.0013534.s001.tif]

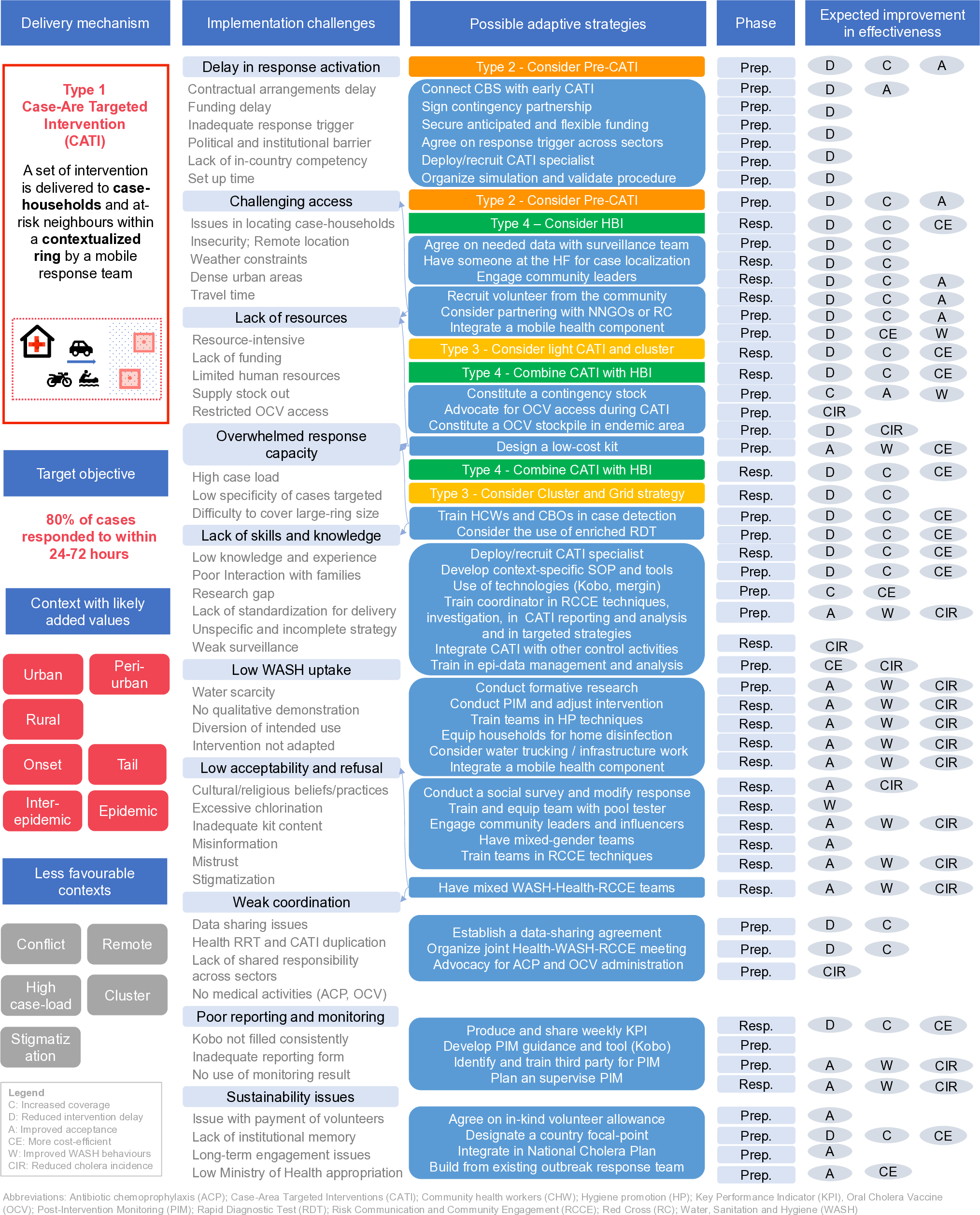

Supplement: S2 Fig — The color-coding system is as follows: red for CATI, orange for pre-CATI, yellow for case-cluster, and green for HBI delivery mechanisms. Adaptive strategies are divided into (1) adaptations to the existing delivery mechanism (blue rectangles) and (2) suggestions to shift or combine with other delivery mechanisms (color rectangles). The figure was created using Wikipedia Commons (https://commons.wikimedia.org/wiki/Accueil). (TIF) [file pntd.0013534.s002.tif]
