# Supplementary material for: Optimizing the implementation of case-area targeted interventions during cholera outbreaks with context-specific delivery mechanisms
Source: PLoS Negl Trop Dis. 2025 Sep 23;19(9):e0013534. doi: 10.1371/journal.pntd.0013534 (PMC12456772; doi:10.1371/journal.pntd.0013534)
Supplement: S1 File — (DOCX) [file pntd.0013534.s006.docx]

Supporting information 1 File. Search rings and results for the narrative review

*Ovid MEDLINE*

- Name of database searched: MEDLINE
- Database platform: Ovid
- Date of search: June 9, 2024
- Timeframe of search: January 01, 2020 to June 7, 2024
- Number of records retrieved: 73

Table A in S1 File: Search ring for Ovid MEDLINE

| **1** | cholera* OR acute watery diahrr* OR vibrio cholerae |
| --- | --- |
| **2** | exp Vibrio Infections/ |
| **3** | 1 OR 2 |
| **4** | ((case-area ADJ3 targeted ADJ3 (intervention? OR response?)) OR (targeted ADJ3 (intervention? OR response?)) OR (ring ADJ3 intervention?) OR ((rapid OR mobile) ADJ3 response) OR (household ADJ3 (intervention? OR response?)) OR (household ADJ3 (disinfection OR spraying)) OR (home ADJ3 disinfection) OR ((health facility-based OR hospital-based) ADJ3 intervention?) OR “alert and response”) |
| **5** | Communicable Disease Control/ |
| **6** | 4 OR 5 |
| **7** | 3 AND 6 |
| **8** | limit 7 to (english language and yr="2020 -Current") |

Table B in S1 File: Search result for Ovid MEDLINE

| ID | Query | Results |
| --- | --- | --- |
| 1 | (cholera* or acute watery diahrr* or vibrio cholerae).mp. | 38,156 |
| 2 | exp Vibrio Infections/ | 13,557 |
| 3 | 1 or 2 | 41,904 |
| 4 | ((case-area adj3 targeted adj3 (intervention? or response?)) or (targeted adj3 (intervention? or response?)) or (ring adj3 intervention?) or ((rapid or mobile) adj3 response) or (household adj3 (intervention? or response?)) or (household adj3 (disinfection or spraying)) or (home adj3 disinfection) or ((health facility-based or hospital-based) adj3 intervention?) or "alert and response").mp. | 50,548 |
| 5 | Communicable Disease Control/ | 33,158 |
| 6 | 4 or 5 | 83,451 |
| 7 | 3 and 6 | 480 |
| 8 | limit 7 to (english language and yr="2020 -Current") | 73 |

*Ovid EMBASE*

- Name of database searched: EMBASE
- Database platform: Ovid
- Date of search: June 9, 2024
- Timeframe of search: January 01, 2020 to June 7, 2024
- Number of records retrieved: 284

Table C in S1 File: Search ring for Ovid EMBASE

| **1** | cholera* OR acute watery diahrr* OR vibrio cholerae |
| --- | --- |
| **2** | cholera/ |
| **3** | 1 OR 2 |
| **4** | ((case-area ADJ3 targeted ADJ3 (intervention? OR response?)) OR (targeted ADJ3 (intervention? OR response?)) OR (ring ADJ3 intervention?) OR ((rapid OR mobile) ADJ3 response) OR (household ADJ3 (intervention? OR response?)) OR (household ADJ3 (disinfection OR spraying)) OR (home ADJ3 disinfection) OR ((health facility-based OR hospital-based) ADJ3 intervention?) OR “alert and response”) |
| **5** | exp communicable disease control/ |
| **6** | 4 OR 5 |
| **7** | 3 AND 6 |
| **8** | limit 7 to (english language and yr="2020 -Current") |

Table D in S1 File: Search result for Ovid EMBASE

| ID | Query | Results |
| --- | --- | --- |
| 1 | (cholera* or acute watery diahrr* or vibrio cholerae).mp. | 48,867 |
| 2 | cholera/ | 14,444 |
| 3 | 1 or 2 | 48,867 |
| 4 | ((case-area adj3 targeted adj3 (intervention? or response?)) or (targeted adj3 (intervention? or response?)) or (ring adj3 intervention?) or ((rapid or mobile) adj3 response) or (household adj3 (intervention? or response?)) or (household adj3 (disinfection or spraying)) or (home adj3 disinfection) or ((health facility-based or hospital-based) adj3 intervention?) or "alert and response").mp. | 70,501 |
| 5 | exp communicable disease control/ | 175,278 |
| 6 | 4 or 5 | 244,572 |
| 7 | 3 and 6 | 1,372 |
| 8 | limit 7 to (english language and yr="2020 -Current") | 284 |
