# Supplementary material for: Optimizing the implementation of case-area targeted interventions during cholera outbreaks with context-specific delivery mechanisms
Source: PLoS Negl Trop Dis. 2025 Sep 23;19(9):e0013534. doi: 10.1371/journal.pntd.0013534 (PMC12456772; doi:10.1371/journal.pntd.0013534)
Supplement: S2 File — (DOCX) [file pntd.0013534.s007.docx]

Supporting information 2 File. Semi-structured interview guide

*Starting the discussion*

Good morning/afternoon, my name is [Name], an MSc student from LSHTM. As you know, we are gathered here today for an hour interview on Case-Area Targeted Interventions (CATI). Before we start, I want to confirm that I've received your signed consent form. Could you verbally confirm your consent? Also, do you have any other questions or concerns about the interview process?

Please be aware that the interview will be recorded / won’t be recorded. Additionally, you have the option to stop the interview at any time.

During the interview, we will explore how Case-Area Targeted Interventions (CATI) can be effectively implemented when dealing with challenges that happen recurrently and are specific to the context. For the purpose of this interview, we define CATI as a set of interventions timely delivered to households with cholera cases and ideally extended to nearby neighbors.

Thank you very much for making yourself available for the interview.

Are you ready to start?

*Roles and range of experience in CATI*

Objective: To gather insights from the interviewee regarding their role, experience, skills and expertise involvement in the CATI approach development.

**Question: Could you tell me a bit about yourself, what you do, and your experience with the CATI approach?**

Prompt:

- Could you share any specific CATI projects you were involved in?
- What was your role?
- In which country(s) and context(s) the CATI projects were implemented?
- What skills and expertise have you developed?

*CATI delivery mechanism description*

Objective: Describe the different CATI delivery mechanisms.

Question(s):

- **Can you share the various CATI delivery mechanisms you have come across, researched or implemented in your experience?**

Questions:

- Can you share the various CATI implementation mechanisms you have encountered, researched, or implemented in your experience?
- What was the context, the cholera situation, the date of the outbreak, and the phase of the epidemic?
- Who led and was involved in the CATI response?
- Which sectors were involved?
- What were the components of the intervention?
- What was the composition of the CATI team and the profile of the team members? How are they trained?
- How was CATI triggered at the beginning of the epidemic and throughout the epidemic? Is an alert system in place?
- How was the intervention carried out?
- What was the size of the ring?
- What were the delay, coverage, and cost of CATI?
- What financing mechanism was put in place?

MSF

- What were the performance and monitoring indicators? What tools?
- Is CATI integrated in National Control Plan or country strategic document?

*Recurrent challenges specific to the context*

Objective: Identify the commonly faced context-specific challenges when implementing CATIs.

**Question: In your experience, what are the main challenges frequently face when implementing CATIs, and in what specific contexts do these challenges arise?**

Prompt:

- Can you provide specific details on the contexts in which these challenges tend to arise?
- How quickly can CATI be implemented as a strategy within the larger cholera response strategy?
- Do you have sufficient surveillance data to target CATI appropriately and quickly?
- Were you able to respond to all cases? In hard-to-reach areas? During outbreak peak?
- Was there any duplication of activities between the Health and WASH sectors?
- Have you encountered any challenges regarding who leads CATI on the ground?
- Have you encountered any challenges in human resources, logistics, or funding when addressing the CATI ring quickly enough?
- Have you noticed different applications of the SOP?
- How is the community receiving the activity, and how effectively is it engaging them?
- Have you observed any challenges in the monitoring and utilization of intervention data?
- Have you seen any challenges in post-intervention monitoring?

*CATI delivery mechanisms limitations and opportunities*

Objective: Describe the different CATI delivery mechanisms, including their limitations and opportunities.

Question(s):

- **Can you share the various CATI delivery mechanisms you have come across, researched or implemented in your experience?**

Prompt:

- The CATI implementation mechanisms may include the use of digital technologies or an adaptation to the standard approach, such as pre-positioning for pre-CATI in conflict zones.
- What limitations have you observed in the CATI implementation mechanisms you have used, and conversely, what opportunities or advantages do these mechanisms offer for effective implementation?
- Have there been any equity issues in the response, where some communities are treated via CATI and others do not receive interventions?
- Does CATI prevent a broader response that can be prepared while the CATIs are being implemented?
- In what context is the implementation mechanism not suitable?
- When do you think we should stop the CATI approach and change strategy? If so, what alternative strategy should we adopt?
- Have you adapted this mechanism according to different contexts?

*Improvements for CATI implementation*

Objective: Explore potential enhancements for the effectiveness of CATI implementation, considering various contexts, as well as the limitations and opportunities associated with different delivery mechanisms.

**Question: In your experience, how do you envision potential enhancements to improve the effectiveness of CATI implementation, considering the diverse contexts and the limitations, and opportunities of the various delivery mechanisms?**

Prompt:

- Take into account the different contexts in which CATIs are implemented and the challenges encountered. Are there any specific adaptations or modifications that you believe could optimize their effectiveness in terms of coverage and response time in various contexts?
- How do you think technological advances could help improve the overall efficiency of CATI implementation?
- Do you see any mechanisms that could reduce the resources (human resources, motivation, logistics, supplies) needed to successfully conduct CATIs?
- Do you think CATI implementation could benefit from community surveillance and oral rehydration points (ORP)?
- Why and how should the link between surveillance and CATI be strengthened?
- Do you think post-intervention monitoring of CATIs should be reinforced and why?

*Documentation*

Objective: To obtain relevant documentation from participants to enhance the depth and accuracy of the information gathered during the interview.

**Question: Could you please share any relevant documentation or materials you have that would provide additional insight into the discussion we had?**

*Interviewee referrals*

Objective: Identify future interviewees I should talk to in order to enrich the CATI implementation framework development process.

**Questions: From our discussion today, are there specific people you would recommend I reach out to?**

Prompt: What do you think that person could contribute to the discussion?

*Conclusion*

Objective: Discuss areas that participants feel were not properly covered before and explore their willingness to receive the final report.

**Questions:**

- **Do you feel to add anything?**
- **Would you like to receive the final report?**
